# Methodological Considerations Regarding Voluntary Slow Breathing Exercise in Hypertensive Populations

**DOI:** 10.1002/clc.70322

**Published:** 2026-04-20

**Authors:** Kaya Oguz Kaan

**Affiliations:** ^1^ TC Saglik Bakanligi Antalya Egitim ve Arastirma Hastanesi Antalya Türkiye

Dear Editor,

We read with great interest the recently published systematic review and meta‐analysis by Cheng et al. in Clinical Cardiology evaluating the effects of voluntary slow breathing exercise on cardiovascular parameters in patients with hypertension [1]. The authors should be commended for addressing a clinically relevant and increasingly utilized non‐pharmacological intervention. Their findings demonstrating reductions in systolic and diastolic blood pressure, as well as heart rate, are consistent with prior literature suggesting a potential role of breathing‐based interventions in cardiovascular regulation.

Nevertheless, several methodological considerations merit further discussion.

First, the definition of “voluntary slow breathing exercise” appears overly broad and may introduce substantial intervention heterogeneity. The included studies encompass a wide range of modalities, including mindfulness meditation, relaxation‐based breathing, yoga breathing, pranayama, pursed‐lip breathing, and music‐guided breathing. Although these approaches share a respiratory component, they differ considerably in terms of cognitive engagement, autonomic activation, and physiological pathways. Previous studies have demonstrated that breathing frequency, pacing, and the presence of biofeedback or behavioral components can significantly influence autonomic outcomes, suggesting that these interventions should not be treated as a single homogeneous entity [2, 3, 4].

Second, there is notable clinical heterogeneity among the included populations and comparators. The analysis combines patients with prehypertension, stage 1 hypertension, essential hypertension, and hypertensive urgency, conditions that differ substantially in pathophysiology and baseline cardiovascular risk. In addition, control conditions vary widely, ranging from usual care and passive controls to active interventions such as health education or relaxation. Such variability may affect the internal validity of pooled estimates and complicates interpretation of the true magnitude of effect. Contemporary hypertension guidelines emphasize individualized and risk‐based management strategies, highlighting the importance of patient stratification in both clinical practice and research [5, 6].

Third, intervention duration varies markedly across the included trials, from acute interventions lasting only minutes to programs extending over several months. Acute reductions in blood pressure or heart rate may reflect transient autonomic responses rather than sustained antihypertensive effects. Previous meta‐analytic evidence has demonstrated a dose–response relationship between total intervention exposure and blood pressure reduction, underscoring the importance of intervention intensity and duration in determining clinical benefit [7].

Furthermore, the conclusions regarding autonomic modulation should be interpreted with caution. The analysis of the LF/HF ratio is based on a limited number of studies and demonstrated instability in sensitivity analyses. In addition, the physiological interpretation of LF/HF as a marker of sympathovagal balance remains debated, further limiting the strength of conclusions related to autonomic function [2, 8].

Despite these limitations, this study contributes valuable evidence supporting the potential role of breathing‐based interventions as adjunctive strategies in hypertension management. Current scientific statements have recognized slow breathing as a promising complementary approach, although further high‐quality, standardized trials are needed before firm clinical recommendations can be established [9]. Importantly, given the simplicity, safety, and low cost of breathing‐based interventions, clarifying their true clinical efficacy remains highly relevant for real‐world hypertension management.

Future research should prioritize standardized intervention protocols, stratified analyses according to hypertension stage, clear separation of acute versus long‐term effects, and incorporation of objective autonomic and hemodynamic biomarkers.

We congratulate the authors for their important contribution and believe that addressing these considerations will enhance the clinical applicability of this emerging therapeutic approach.

## Conflicts of Interest

The author declares no conflicts of interest.

## Data Availability

Data sharing not applicable to this article as no datasets were generated or analyzed during the current study.
